# Hard to Swallow: Scaling Relationships Between the Size of Avian Prey and the Overall Size and Maximal Gape of Brown Treesnakes

**DOI:** 10.1002/ece3.71338

**Published:** 2025-04-21

**Authors:** Shane R. Siers, Juan‐Carlos Mungaray, Martin Kastner, Bruce C. Jayne

**Affiliations:** ^1^ United States Department of Agriculture, Wildlife Services National Wildlife Research Center Barrigada Guam; ^2^ Research Corporation of the University of Guam University of Guam Mangilao Guam; ^3^ Department of Fish and Wildlife Conservation Virginia Tech Blacksburg Virginia USA; ^4^ Department of Biological Sciences University of Cincinnati Cincinnati Ohio USA

**Keywords:** allometry, behavior, bird, feeding ecology, predation, snake

## Abstract

Snakes are a useful model for gaining insights into the relationships between predator and prey sizes and resource utilization because their anatomy limits the size of prey that can be swallowed whole. However, data are sparse regarding how commonly gape‐limited predators eat or attempt to eat prey with sizes up to or exceeding their maximal gape. Thus, for an invasive predator, the brown treesnake ( *Boiga irregularis* ), we fed captive snakes dead birds with an extremely large range (17%–447%) of relative prey area (RPA = prey cross‐sectional area/snake gape area) to test the predictive value of RPA for snakes attempting to ingest or successfully ingesting prey. As expected, RPA significantly predicted (logistic regression *p* < 0.0001) the probability of birds being eaten, with an upper size limit similar to the maximal gape of the snakes. Although RPA also significantly predicted (*p* = 0.003) the probability of attempting to eat a bird, it was less accurate in predicting attempts than successes, and many snakes attempted to eat birds too large to swallow. Twenty‐five snakes attempted to eat birds with RPA ranging from 130% to 447%. The longest durations of unsuccessful feeding attempts were often for values of RPA near 100% rather than the extremely large values. For six large birds with mean measured RPA = 93%, the prey diameter soon after ingestion averaged 14% less than that measured prior to ingestion, which can allow snakes to consume 30% more mass than would otherwise be possible. Our findings complement a recent field study that concluded brown treesnakes regularly attempt to eat live birds too large to swallow. Our results also greatly expanded the known range of avian prey sizes that these snakes attempt to eat. Consequently, brown treesnakes pose a risk to birds with sizes well beyond the limit on prey size imposed by gape.

## Graphical abstract

The anatomy of snakes and other gape‐limited predators imposes an upper limit on the size of prey that are swallowed whole, and maximal gape was a good predictor of the maximal size of prey eaten by the invasive brown treesnakes in our study. However, gape had modest predictive value for the size of prey that snakes attempted to eat as some attempts involved birds more than four times larger than what could be swallowed. Hence, the threat of brown treesnakes to avian prey is far greater than just the size of birds small enough to be eaten.

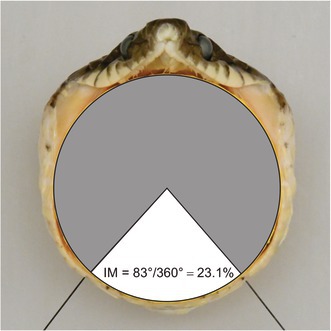

## 1 Introduction

The size of prey relative to that of a predator has important consequences for the energy that a predator can gain from prey and whether a predator can subdue, kill, or consume prey, which in turn can affect prey populations. Diverse taxa of vertebrate predators, including many species of fishes (Wainwright and Richard 1995), amphibians (Emerson 1985), reptiles (Shine and Thomas 2005), and birds (Wheelwright 1985), often consume their food items whole with little alteration of shape; hence, the maximal size of the mouth opening (gape) can impose an anatomical limit of prey size. Within vertebrates, and especially terrestrial vertebrates, snakes have evolved several unique anatomical specializations that allow them to swallow spectacularly large prey (Cundall and Irish 2022).

Although the anatomy of gape‐limited predators, such as snakes, clearly imposes an upper limit on the size of prey that are consumed, other factors may be equally important for influencing prey size. For example, even when a prey item is small enough to swallow, a snake may simply choose not to eat it (Gripshover and Jayne 2023). Additionally, formidable anatomical, chemical, or behavioral defensive mechanisms of the prey may impede predation or limit prey size (Kornilev et al. 2022). For instance, in nature, some species of snakes that specialize in hard‐shelled crustaceans only eat prey substantially less than their maximal gape, whereas other species that eat crustaceans that are soft and largely defenseless shortly after molting often do eat prey near the maximal size permitted by their gape (Gripshover and Jayne 2021, 2023; Jayne et al. 2018).

The effects of predators on prey populations are not limited only to those arising from eating prey because a predator could also attack or kill prey without eating it. Even if a predator attacked prey without directly killing it, injuries sustained from an attack could ultimately contribute to the death of the prey or be detrimental to its fitness. Although a wealth of studies have documented the sizes of prey eaten by snakes (reviewed in Arnold 1993), only a few recent studies have quantified the size of prey relative to the limits imposed by the gape of snakes (Gripshover and Jayne 2021, 2023; Jayne et al. 2018). Furthermore, data are even more sparse and often anecdotal regarding attacks and attempts of snakes to eat prey that are too large for them to swallow (Natusch et al. 2021), and the usual lack of accompanying direct measurements of gape can obscure whether some failed predation attempts by snakes are indeed because the prey item was too large to swallow.

Two striking examples of how predation by snakes can devastate the native prey populations are the invasive Burmese pythons in Florida (Hoyer et al. 2017) and brown treesnakes in Guam (Savidge 1987). The sizes of these snakes and the species with which they interact can have profound importance, as illustrated by disparities in the sizes of alligators and Burmese pythons being able to reverse which species is the predator and which species is prey (Guzy et al. 2023). More commonly, increased prey size just impedes or prevents consumption by gape‐limited predators, and a recent study of these two invasive species has quantified how the variation in size within and between these species could limit the sizes of different types of prey that are consumed (Jayne et al. 2022). Brown treesnakes in the wild in Guam may often attack and unsuccessfully attempt to eat birds (Kastner et al. 2024). However, this study, which was based on bird carcasses that were partially covered with saliva, was not able to determine the sizes of the snakes responsible for this, and these carcasses were small enough to be eaten by very large brown treesnakes. Thus, despite such recent data for these two important invasive species, data remain very sparse regarding the sizes of prey relative to the gape of snakes that are willing to attack and attempt to eat them.

In this study, we focused on brown treesnakes and used recently refined methods for measuring maximal gape for a large sample ranging from small juveniles to large adults. We used the scaling relationships between snake size and maximal gape to estimate the size of prey items relative to the anatomical limit on prey size. To test the probability that brown treesnakes would attempt to eat prey too large to be swallowed, we offered snakes prey with such a large range in size that some were too large to be swallowed. This facilitates determining the extent to which this invasive species poses a threat to native birds and other fauna beyond those that are small enough to be eaten.

## 2 Methods

### 2.1 Morphology

**FIGURE 1:**
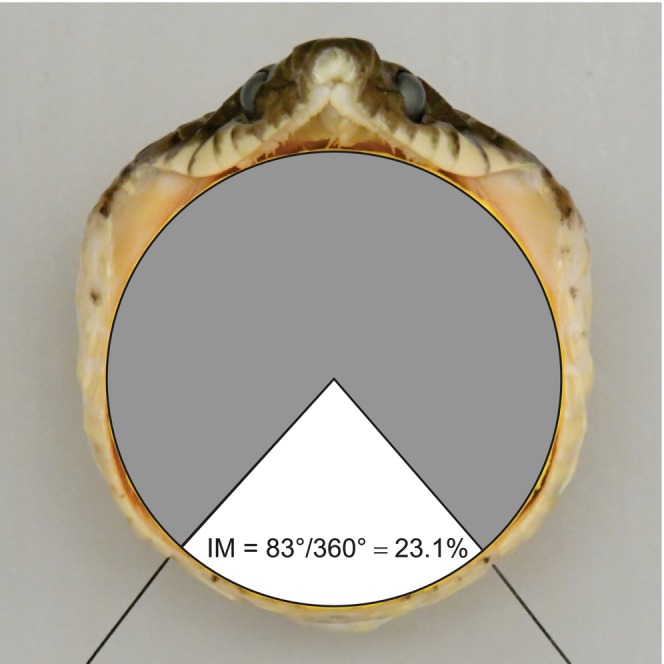
Method for calculating the contribution of intermandibular soft tissues (IM) to maximal gape area. The thin black pins mark the distal ends of the left and right lower jaws. This large adult male had SVL = 169 cm and a maximum gape diameter = 5.9 cm.

For all snakes used in the study, we measured snout‐vent length (SVL), skull length, and mass. We measured gape directly for 230 individuals, including 42 individuals that were not used in feeding trials. We measured gape using the same overall procedures as in recent studies (Gripshover and Jayne 2021, 2023; Jayne 2023). Whenever possible, we measured gape immediately after a specimen was euthanized. Otherwise, after euthanasia, we stored frozen specimens that were covered with water within plastic bags . This method prevented desiccation of the soft tissues and does not significantly affect measurements of gape compared to freshly euthanized specimens (Jayne et al. 2022). For each freshly euthanized or thawed, frozen specimen, we inserted the hemispherical ends of successively larger 3D printed plastic probes with cylindrical shafts into the mouth of the snake. To simulate the lubrication of snake saliva, we coated the probes with a water‐based lubricating jelly. We inserted successively larger probes until a probe was too large to fit or caused the beginning of tissue failure. When appreciable resistance to a probe was encountered, we waited at least 10 min before attempting to insert the next larger probe. Maximal gape diameter (Gdiam) was the size of the largest probe that fit without damaging tissues, and specimens were stored with a cylindrical spacer with a diameter equal to that of maximal gape (Figure 1). The incremental increases in cylinder diameter between successively larger probes were 1, 2, and 3 mm for probes with diameters of < 22, 22–44, and 44–68 mm, respectively. These methods improve upon a previous study quantifying the gape of brown treesnakes (Jayne et al. 2022), which: (1) used probes with an end that had a distinct edge associated with a 45° bevel, (2) used water rather than jelly as a lubricant, and (3) lacked a pause of at least 10 min between the insertion of successively larger probes after appreciable resistance was encountered.

We calculated maximal gape area (Garea) as the area of a circle with a diameter equal to that of Gdiam. As in previous studies (Jayne et al. 2022) and shown in Figure 1, we used anterior view photographs to determine the percentage of Garea arising from the distention of the intermandibular soft tissues between the tips of the lower jaws (IM).

For feeding trials, we standardized a size series of domesticated chicken (*Gallus domesticus*). For prey items with cross‐sectional areas smaller than chicken hatchlings, we used Japanese quail ( *Coturnix japonica* ), having roughly the same body form as chickens. For all prey items, we measured mass and maximal circular cross‐sectional areas using the methods of Jayne et al. (2022). In brief, we placed dead prey headfirst into successively smaller 3D printed funnels with cylindrical stems that were longer than the body of the prey. We then held the funnel vertically and shook it so that the prey slid through. We used successively smaller funnels until the prey item was too large to slide through the stem of the funnel. We estimated prey cross‐sectional area from a circle with a diameter equal to the mean internal diameter of the stems of the last two funnels that were used. The incremental changes in diameter between successively smaller funnels were 10, 5, 4, 3, 2, and 1 mm for funnels with diameters of > 107, 107–77, 77–44, 44–22, and < 22 mm, respectively.

Many of the chickens and quail used in the feeding experiments had identical masses. Consequently, to calculate the scaling relationships of mass relative to area for chickens and quail, we used only a subset of these birds so that no more than three birds within a species had the same mass, and we included some additional specimens that were not offered to snakes to increase the variability in prey size. The resulting sample sizes used for the scaling relationships were 186 for chickens and 56 for Japanese quail.

To gain further insights regarding the size and shape of birds beyond those fed to the snakes in experiments and to facilitate estimating the cross‐sectional area from the masses of passerine species for which direct measurements of area were not possible, we used the same morphometric methods as previously described for 28 passerine birds (Table S1) that were collected after they fatally struck buildings on the campus of the University of Cincinnati. For this sample, with masses ranging from 6.5 to 82 g, we measured one or two specimens per species for a total of 18 species belonging to 13 genera. In Guam, we directly measured the mass and area of 18 black drongos ( *Dicrurus macrocercus* ) from hatchlings to adults with masses ranging from 5.5 to 63 g, and we directly measured mass and diameter for adults of seven additional species representing five orders. These birds were euthanized and received from USDA Wildlife Services animal damage management operations associated with airports and seaports on Guam.

Finally, we tabulated some size data from the literature for bird species found in Guam and the nearby Mariana Islands in the western Pacific Ocean (Table S2). The bird species listed in Table S2 do not include all the species found on Guam, but they were selected to represent the range of sizes (e.g., swiftlet to frigatebird), species driven extinct or extirpated from Guam by brown treesnake predation (e.g., Guam flycatcher, Guam kingfisher, Guam rail, Mariana crow), remaining species of conservation concern (e.g., Mariana swiftlet and Micronesian starling), or species on neighboring islands at risk of brown treesnake introduction (e.g., Rota white‐eye, Tinian monarch).

### 2.2 Feeding Experiments

We conducted a total of 278 feeding trials with a total of 216 individuals, for which all but 10 were wild‐caught individuals that were tested in Guam within 14 days of being captured. Because of the difficulty of obtaining larger snakes (> 120 cm SVL), we conducted three trials per individual for each of 31 large individuals, which included 10 long‐term (> 10 year) captives that were tested at the University of Cincinnati. We waited a minimum of 7 days between successive trials when more than one trial per individual was conducted. The prey items for all trials were either dead Japanese quail or chickens that had been frozen and then warmed to 32°C–35°C before being placed in the container with the snake. All experiments and procedures were in compliance with the Institutional Animal Care and Use Committees of the University of Cincinnati (protocol 21‐05‐26‐02) and the USDA National Wildlife Research Center (QA‐3374).

Before the feeding trials, we used the scaling equation of Jayne et al. (2022) to estimate the maximal gape of each snake based on its SVL. We attempted to offer prey with a wide variety of directly measured cross‐sectional areas that had predicted values of relative prey area (RPA = prey area/gape area) ranging relatively evenly from approximately 35% to 150% RPA, bracketing the approximate anatomical limit on prey size (100%), as well as several predicted values well above this size (150%–446%). After the feeding trials, the snakes were euthanized, and we were able to measure gape directly for 178 of the individuals used in the feeding trials. Whenever possible for subsequent analyses, we recalculated RPA based on directly measured gape. For the remainder of the individuals for which direct measurement was not possible, we recalculated RPA using a value of maximal gape estimated from SVL using the scaling equations derived from the 230 direct measurements of gape in the present study.

For the trials in Guam, snakes were placed in plastic storage bins modified with ample ventilation and a clear plastic pane for viewing and light–dark diel cycle cues. Feeding chambers for snakes ≤ 120 cm were 64 L in volume (floor 57 × 34 cm at the bottom, longer and wider at the top; height = 28 cm) and snakes > 120 cm were held in similar but larger 102‐L tubs (floor 60 × 37 cm; height = 33 cm). To record ingestion attempts, a small battery‐operated infrared trail camera was affixed to the inside of the lid of each feeding chamber and set to record one image every minute during the entire feeding trial. Feeding chambers were maintained within an animal testing facility at the Wildlife Services compound in Barrigada, Guam. Temperatures were maintained at 24°C–25°C to be similar to Guam's ambient temperatures. Each snake was acclimatized to its feeding chamber for 3 nights prior to the feeding trials. For the trials at the University of Cincinnati, we videotaped (1 image/s) feedings of each snake in its own permanent cage (floor 34 × 75 cm; height = 55 cm), which had an incandescent light bulb (12 h light:12 h dark) that allowed behavioral thermoregulation of body temperatures of up to 32°C during the daytime when the trials were conducted, while the ambient room temperature was a constant 27°C.

Each trial began when we offered a directly measured, dead, thawed prey item that was left for the snake to attempt to ingest at will, and prey items remained available to the snake for two nights. If the prey was not taken after the second night, the trial was terminated. If the snake was in the process of ingestion, or had fully ingested the prey item, we monitored for an additional 24 h to observe the completion of ingestion or regurgitation. For each trial, we reviewed the camera images and recorded whether or not the snake attempted to feed, and we scored each attempt within a trial with a value of 1–5 as follows: (1) the snake bit the prey but failed to swallow any part of the prey so that it was posterior to the head of the snake; (2) only the head and neck or a foot of the prey was swallowed; (3) swallowing progressed to more than just a foot or the head and neck but not to the thickest part of the body; (4) swallowing was not complete, but the snout of the snake had progressed to the thickest part of the body; or (5) the prey item was completely ingested. We recorded the duration (minutes) of each attempt and, when a trial had more than one attempt as a result of the snake completely releasing the prey and then starting again, we recorded the total duration of all attempts and the greatest amount of swallowing that occurred in any of the attempts within a trial (maxscore). We also recorded all trials in which the prey were completely swallowed but subsequently regurgitated.

### 2.3 Data Analysis

To calculate the scaling relationships between different morphological variables, all data were log_10_ transformed and analyzed with an ordinary least squares (Kilmer and Rodriguez 2016) linear regression (log*Y* = log*b* + a*log*X*), which is equivalent to the equation *Y* = *bX* ^*a*^ where *a* is the scaling exponent (Huxley and Teissier 1936). Geometrically similar individuals would have isometric scaling when all lengths (*L*) scale with other lengths with an exponent of 1, but positive and negative allometry refer to scaling exponents more or less than 1, respectively (Huxley and Teissier 1936). Thus, with geometric similarity, areas and volumes (masses) scale with *L* ^2^ and *L* ^3^, respectively; hence, area scales with mass^2/3^ (Table 1, column 3). To test whether the calculated slopes differed significantly from those expected from geometric similarity (isometry), we examined whether or not the value expected from isometry was within the 95% CL of the calculated slope for the regression.

**TABLE 1:** Least squares regression statistics (±95% CL) for the scaling equations of brown treesnake morphology.

Variables		Exp	This study *N* = 230				Jayne et al. (2022) *N* = 19			
Ind	Dep		Obs	Slope	Intercept	*R* ^2^	Obs	Slope	Intercept	*R* ^2^
SVL	Mass	3	+	3.594 ± 0.113	−5.146 ± 0.223	0.945	+	3.398 ± 0.283	−4.684 ± 0.560	0.974
SVL	Gdiam	1	+	1.154 ± 0.042	−1.838 ± 0.084	0.927	=	1.009 ± 0.113	−1.608 ± 0.225	0.954
SVL	Garea	2	+	2.310 ± 0.085	−3.780 ± 0.167	0.927	=	2.017 ± 0.227	−3.320 ± 0.449	0.954
SVL	SKL	1	−	0.622 ± 0.037	−0.890 ± 0.073	0.831	−	0.668 ± 0.069	−0.983 ± 0.136	0.961
SKL	Garea	2	+	3.098 ± 0.217	−0.268 ± 0.075	0.777	+	3.000 ± 0.215	−0.348 ± 0.077	0.981
Mass	Garea	0.67	−	0.613 ± 0.028	−0.416 ± 0.055	0.892	−	0.588 ± 0.061	−0.529 ± 0.130	0.961
SVL^a^	Gdiam (*N* = 110)	1	+	1.151 ± 0.052	−1.800 ± 0.099	0.950				
SVL^b^	Gdiam (*N* = 45)	1	+	1.110 ± 0.051	−1.692 ± 0.106	0.977				

*Note:* Independent and dependent variables are indicated by Ind and Dep, respectively. The slopes expected from geometric similarity are indicated by Exp. Observed slopes (Obs) that conformed to isometry (based on 95% CL) or had negative or positive allometry are indicated by =, − and +, respectively. All *p*‐values for the test of the overall significance of the regression (slope not equal to 0) were less than 10^−8^. Units of distance, area and mass are cm, cm^2^ and g, respectively, and all values were log_10_ transformed.

Abbreviations: Garea, maximal gape area; Gdiam, maximal gape diameter; SKL, skull length; SVL, snout‐vent length.

^a^ A subset of individuals excluding those with negative residual values of Gdiam predicted from the regression with the entire sample of 230 individuals.

^b^ A subset of individuals excluding those with negative residual values of Gdiam predicted from the regression with the sample of 110 individuals.

One key goal for this study was to determine how likely a snake of a particular size would be to have a gape sufficiently large to eat a bird of a particular size. Of course, individual snakes have values of maximal gape both greater and less than the value predicted from the least squares regression using a metric of overall snake size. Consequently, for the least squares regressions, we also calculated the 95% prediction limits for a single observation (Sokal and Rohlf 1981, box 14.3). Furthermore, we also estimated the upper boundary Gdiam versus SVL with the same method as described in King (2002) in which successive regression analyses only use a subset of data with values of the dependent variable that are above the regression line (positive residuals) from a preceding regression analysis.

To test the predictive value of relative prey area (RPA) for dependent variables indicating whether or not snakes attempted to eat or ate prey, we used Systat version 9 to calculate logistic regressions in which the independent variable was RPA. For any trials in which the snake attempted to eat prey or ate the prey, the dependent variable “try” was assigned a value of 1, whereas a value of 0 indicated that no attempt of any kind was made. The dependent variable “eat” was assigned a value of 1 for all trials when prey were completely eaten and a value of 0 for all other trials.

## 3 Results

### 3.1 Morphology

**FIGURE 2:**
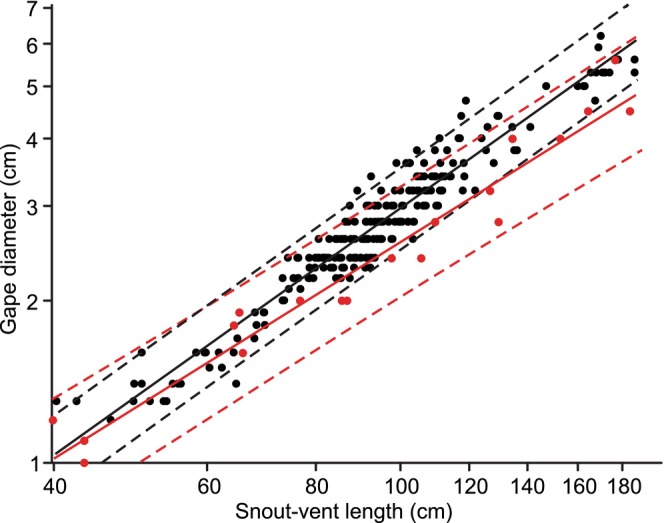
Log–log plots of the scaling relationships between maximal gape diameter and snout‐vent length. Black (*N* = 230) and red (*N* = 19) are the data for this study and (Jayne et al. 2022), respectively. See Table 1 for the regression statistics.

Despite the length of the skull having negative allometry with SVL, maximal gape (diameter and area) had positive allometry with SVL (Table 1; Figure 2). The masses of the snakes had strong positive allometry with SVL, which contributed to maximal gape area having negative allometry with snake mass (Table 1). The contribution of the intermandibular soft tissues (IM) to gape area increased significantly with increased SVL (IM = 16.32*logSVL—13.31, *R* ^2^ = 0.31, *p* < 0.001, *N* = 230).

**FIGURE 3:**
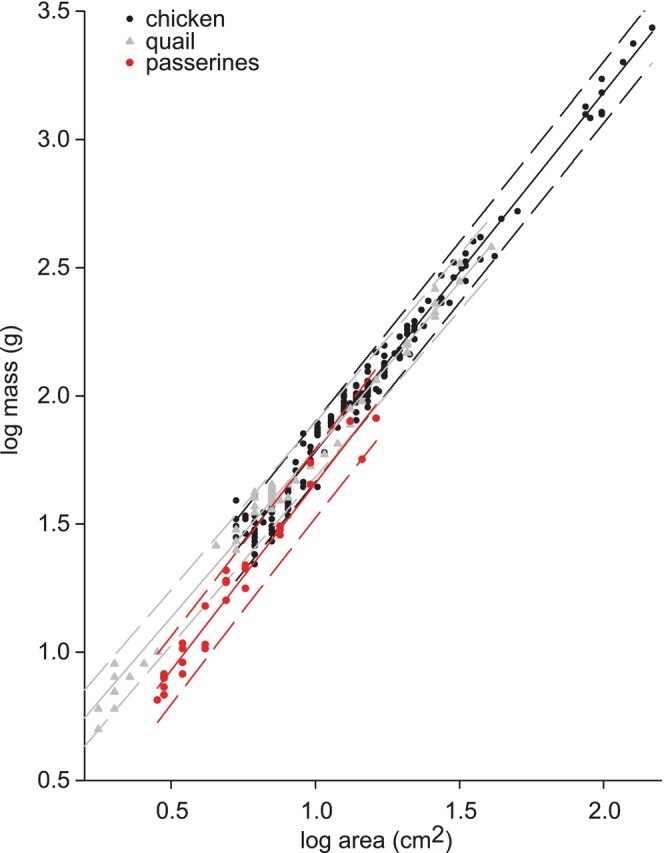
Scaling relationships for mass and cross‐sectional area of birds. The solid lines are the values predicted for least squares regressions, and the dashed lines are the 95% prediction limits. See Table 2 for the regression statistics, and see Table S1 for the North American species used for the passerine data.

For three of the four observed taxonomic groups of birds, the slopes of mass predicted from cross‐sectional area were slightly but significantly less than the value of 1.5 expected from isometry (Table 2). The slope of the regression for chickens was significantly greater than that for quail (ANCOVA *F*_1,238_ = 14.8; *p* = 0.00016). However, because the regression lines for chickens and quail crossed, many of the predicted values of mass for a given cross‐sectional area were extremely similar for intermediate values of cross‐sectional area (Figure 3). By contrast, the slopes for mass versus area (Figure 3) did not differ significantly either between chickens and black drongos (ANCOVA *F*_1,200_ = 0.1; *p* = 0.74) or between chickens and other Passeriformes (ANCOVA *F*_1,210_ = 1.51; *p* = 0.28), but the masses of chickens for a given area were significantly greater than those of both black drongos (ANCOVA *F*_1,201_ = 231; *p* < 0.001) and other passerines (ANCOVA *F*_1,211_ = 3.82; *p* < 0.001).

**TABLE 2:** Least squares regression statistics (±95% CL) for the scaling equations of bird mass versus cross‐sectional area.

Taxon	*N*	Slope	Intercept	*R* ^2^
Chicken	186	1.400 ± 0.014	0.382 ± 0.017	0.982
Quail	56	1.308 ± 0.018	0.482 ± 0.017	0.990
Passeriformes	28	1.458 ± 0.054	0.200 ± 0.040	0.966
Drongo	18	1.379 ± 0.072	0.178 ± 0.074	0.958

*Note:* All *p*‐values for the test of the overall significance of the regression (slope not equal to 0) were less than 10^−8^. Units of area and mass are cm^2^ and g, respectively, and all values were log_10_ transformed.

The combined scaling relationships for brown treesnakes and chickens can predict the maximal mass of chickens that could be eaten by brown treesnakes with a given SVL (Table 3). For example, the upper 95% prediction of gape diameter for a very small juvenile snake (SVL = 40 cm; mass = 4.1 g) is 1.23 cm, which would accommodate a 3.2‐g bird with the shape of a chicken, whereas for an adult snake with SVL = 120 cm and mass = 212 g, the corresponding values of gape diameter and chicken mass are 4.35 cm and 111 g, respectively. For these two examples, the corresponding values of relative prey mass (RPM) are 78% and 52%, respectively. Hence, as a result of the scaling relationships, the relative prey mass that can be eaten decreases substantially with increased snake size (Table 3).

**TABLE 3:** Predicted values of maximal gape diameter (cm) and chicken mass at maximal gape diameter for snakes of a given SVL and mass (predicted for SVL).

Snake		This study (230 snakes)				Jayne et al. (2022) (19 snakes)			
		Gdiam (cm)		Bird mass (g)		Gdiam (cm)		Bird mass (g)	
SVL (cm)	Mass (g)	Mean	Upper 95% prediction	Mean	Upper 95% prediction	Mean	Upper 95% prediction	Mean	Upper 95% prediction
40	4.1	1.03	1.23	1.9	3.2	1.02	1.32	1.9	3.9
60	17.6	1.64	1.95	7.1	11.6	1.54	1.95	6.0	11.6
80	49.4	2.29	2.72	18.2	29.6	2.05	2.60	13.4	26.1
100	110	2.97	3.52	37.9	61.0	2.57	3.25	25.2	48.8
120	212	3.66	4.35	68.1	110.6	3.09	3.92	42.3	82.5
140	369	4.37	5.20	112.0	182.5	3.61	4.59	65.5	128.6
160	596	5.10	6.07	172.8	281.8	4.13	5.27	95.6	189.5
180	911	5.85	6.96	254.0	413.8	4.65	5.95	133.3	266.4

### 3.2 Feeding Experiments

**FIGURE 4:**
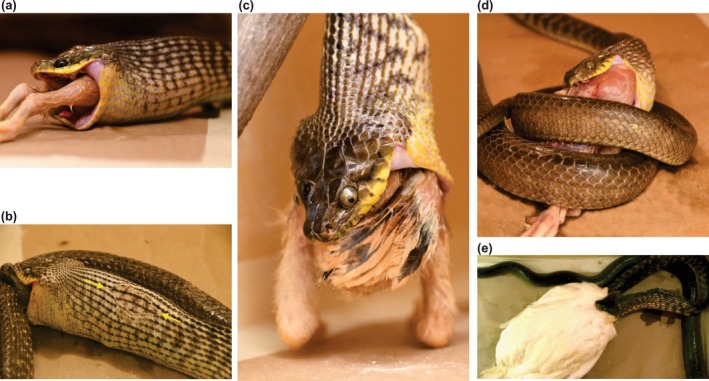
Large, long‐term captive brown treesnakes eating (a–c) or attempting to eat (d, e) large chickens. (a) Note the copious amount of saliva coating the feathers. Prey RPA = 88% and 108 g, and snake SVL = 119 cm and mass = 620 g. (b) Rupture of the outer most layer of skin (between arrows) during ingestion. Prey RPA = 95% and mass = 230 g, and snake SVL = 170 cm and mass = 1290 g. (c) The largest relative prey size eaten during the feeding trials. Prey RPA = 108% and mass = 187 g, and snake SVL = 168 cm and mass = 1210 g. (d) The longest feeding attempt. Prey RPA = 152% and mass 340 g, and snake SVL = 169 and mass = 900 g. Before this feeding trial the chicken had diameter of 6.9 cm, but after the snake repeatedly used its coils to squeeze the chicken while attempting to swallow it, the prey diameter was 5.5 cm. (e) The largest prey item that a snake attempted to eat during the feeding trials. Prey RPA = 447% and mass 1250 g, and snake SVL = 186 cm and mass = 1160 g. Although the snake completely swallowed the head and neck of the chicken (e), it reached an impasse at the shoulders of the bird (maxscore = 3). For a–e the total durations of ingestion or the feeding attempts were 20, 110, 182, 417, and 21 min, respectively.

For the total of 278 feeding trials and all sizes of prey, approximately one‐half of the trials ended with the snakes not attempting to eat the prey (Table 4). For all trials with prey with RPA < 100%, slightly more than one‐third of the prey were completely refused, but attempts without successful ingestion were rare (Table 4). The percentage of trials with complete refusals was substantially greater for recently caught snakes (54%) than for the long‐term captives (10%). Furthermore, the only (3) trials with the long‐term captives that ended with complete refusals had values of RPA of 152%, 213%, and 447%. Both the recently caught and long‐term captives had many unsuccessful attempts to eat prey larger than their gape, but the percentages of trials with attempts to eat categories of prey with RPA > 100% were nearly five times greater for the long‐term captives compared to the recently caught snakes (Table 3). The five largest values of RPA for prey eaten by snakes (114%, 129%, 131%, 133% and 149%) were all for wild‐caught snakes with estimated values of gape (not directly measured postmortem), whereas the largest value of RPA for a prey item that was eaten by a snake with directly measured gape was 110%. Remarkably, at least some snakes attempted to eat prey over the entire range of prey size offered, which included an extraordinarily large maximum value of RPA of 447% (Figure 4e).

**TABLE 4:** Outcomes of feeding trials based on relative prey area (RPA) and different subsamples.

	RPA				Total
	< 100%	101%–125%	126%–150%	> 150%	
*All*					
*N*	137	47	37	57	278
%refuse	35.0	57.4	62.2	70.2	49.6
%try only	5.8	27.7	27.0	29.8	17.3
%eat	59.1	14.9	10.8	0	33.1
*Obsgape*					
*N*	97	35	19	57	208
%refuse	33.0	54.3	84.2	70.2	51.4
%try only	5.2	31.4	0	29.8	15.9
%eat	61.9	14.3	15.8	0	32.7
*Wild*					
*N*	127	41	34	46	248
%refuse	37.8	65.9	67.6	80.4	54.4
%try only	6.3	22.0	20.6	19.6	13.3
%eat	55.9	12.2	11.8	0	32.3
*Captive*					
*N*	10	6	3	11	30
%refuse	0	0	0	27.3	10.0
%try only	0	66.7	100	72.7	50.0
%eat	100	33.3	0	0	40.0

*Note:* The four different samples in different rows consisted of: (1) all feeding trials, (2) only trials with snakes for which gape was measured directly (Obsgape), (3) wild‐caught individuals (wild), and (4) long‐term captives (captive). The outcomes of feeding trials were: (1) a complete refusal to attempt to eat (refuse), (2) any attempt to eat in which the prey was not eaten (try only), and (3) trials in which prey were eaten.

**FIGURE 5:**
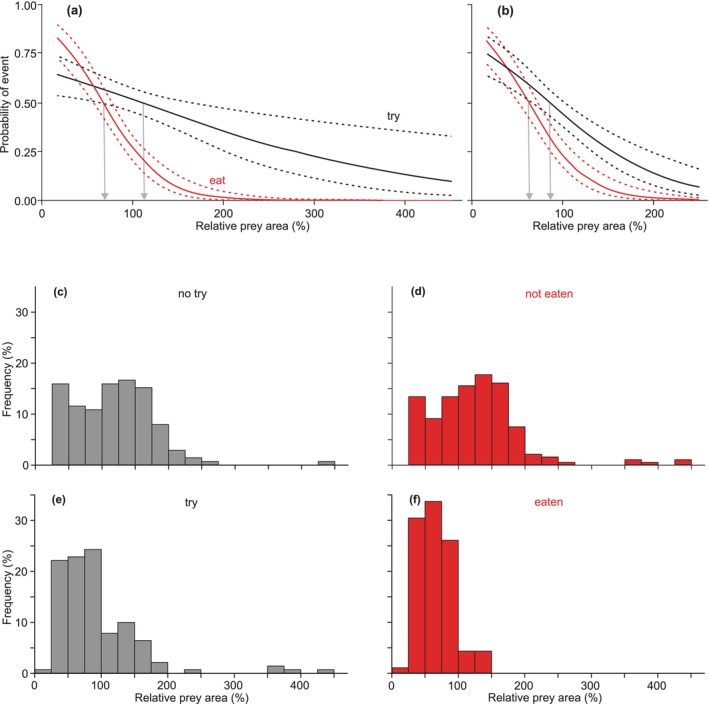
Probabilities and frequencies of snake feeding behaviors. (a, b) Logistic regressions predicting the probability of attempting to eat (try) or successfully eating birds based on the values of RPA of the prey used in laboratory feeding trials. (a) Regressions for all 278 trials. (b) Regressions only for recently caught snakes tested in Guam (*N* = 248). The solid lines are the values predicted from the regressions, and the dashed lines are the 95% prediction limits. The gray arrows indicate the values of RPA when the predicted probability was 0.5. (c, d) Frequency distributions for the samples in panel a. (c) *N* = 138. (d) *N* = 186. (e) *N* = 140. (f) *N* = 92.

Logistic regressions for all 278 feeding trials revealed that RPA had highly significant predictive value for whether or not a snake attempted to eat prey (*p* = 0.003) and whether a snake successfully ate prey (*p* < 0.0001). The values of RPA at a probability of 0.5 for whether any attempt occurred and for whether prey was eaten were 108% and 67%, respectively (Figure 5a), and the logistic regressions correctly predicted the outcomes in these two analyses for 52% and 68% of the cases, respectively. For the entire sample (Figure 5a), the snakes did (Figure 5e) or did not attempt (Figure 5c) to eat the prey with pre‐ingestion sizes exceeding their maximal gape (RPA = 100%) in 62% and 30% of the trials, respectively, whereas only 9% of the prey that were eaten (Figure 5f) had RPA > 100%. Restricting the sample to only the recently‐caught snakes had little effect on the logistic regression for the probability of prey being eaten, but the curve predicting whether or not snakes would attempt to eat the prey shifted downward and to the left considerably (Figure 5b). Restricting the sample to snakes with directly‐measured gape did not appreciably change the logistic regressions compared to those for the entire sample. For the long‐term captives, the logistic regression predicting an attempt based on RPA was not statistically significant (*p* = 0.13) even though it correctly predicted 84% of the outcomes. For the long‐term captives, the logistic regression predicting whether or not snakes ate was not quite significant (*p* = 0.060), but it correctly predicted 90% of the outcomes, and the value of RPA at a probability of 0.5 was 103%.

**FIGURE 6:**
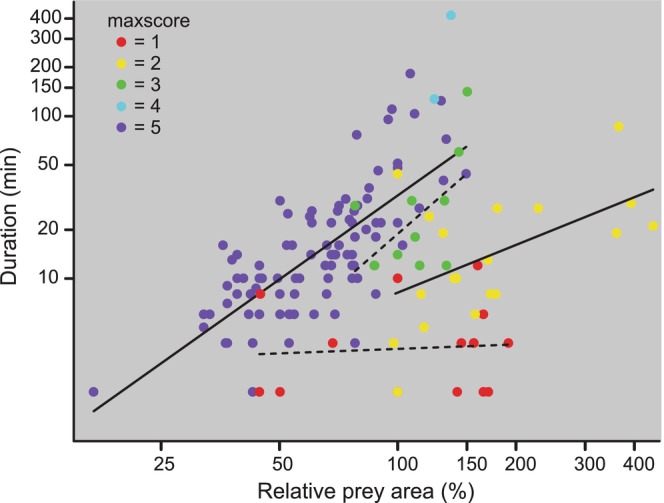
Log–log plots of total attempt duration per trial versus relative prey area. When snakes ate the prey (maxscore = 5), the duration of the attempt increased significantly with increased RPA (log(duration) = 1.717*log(RPA) − 1.922, *R* ^2^ = 0.548, *p* < 0.001, *N* = 92). However, for snakes that did not eat the prey, progressively greater departures from completely engulfing the prey decreased the correlation between attempt duration and RPA (maxscore = 3, *R* ^2^ = 0.329, *p* = 0.065, *N* = 11; maxscore = 2 *R* ^2^ = 0.290, *p* = 0.014, *N* = 20; maxscore = 1 *R* ^2^ = 0.006, *p* = 0.786, *N* = 15). Values of maxscore of 1, 2, 3, and 4 indicate: Prey bitten but no swallowing, swallowing of only the head and neck or a foot, and swallowing of more than the head and neck or a foot but not swallowing up to the thickest part of the prey, and the snout of the snake during swallowing progressing up to the thickest part of the prey. The solid and dashed lines indicate significant and non‐significant least squares regressions, respectively.

When a feeding attempt was made, the amount of the prey engulfed by the snakes varied substantially among the trials and depended on relative prey size. Of the total of 140 trials that had at least one attempt, values of maxscore of 1, 2, 3, 4, and 5 (eaten) were observed in 15, 20, 11, 2, and 92 trials, respectively (Figure 6). For the trials with values of maxscore of 1, 2, 3, 4, and 5, the ranges in RPA were 44%–191%, 98%–447%, 87%–150%, 124%–137%, and 17%–149%, respectively. A prey item was regurgitated after being completely swallowed in only one trial (RPA = 124%).

The total duration of feeding attempts increased significantly with increased RPA in the 92 trials when prey were eaten (Figure 6). However, as the amount of prey engulfed decreased, the predictive value (*R* ^2^) of RPA for the total duration of the attempt in trials progressively decreased and became non‐significant (Figure 6). For the 17 trials with an attempt when RPA > 150%, none of the prey were eaten, and all but one (86 min) of these trials had attempt durations between 2 and 29 min (Figure 6). The eight trials with the longest durations of the feeding attempts had values of RPA between 95% and 150% (Figure 6). Thus, many snakes were most persistent in attempting to eat prey that had sizes reasonably near the maximal gape of the snake.

Seven of the long‐term captives ate large chickens (mean RPA = 93%) with pre‐ingestion diameters of 4.3, 4.4, 5.2, 5.2, 5.5, 5.9, and 6.1 cm, but when these prey were recovered from the stomachs of the snakes and re‐measured, the post‐ingestion diameters were 3.7, 3.7, 4.9, 4.1, 4.7, 5.8, and 4.6 cm, respectively. Hence, the ingestion process reduced the circular diameter of these large prey by an average of 14% even though snakes only coiled forcibly around the prey in the last of these feedings. One feeding attempt by a large snake that coiled repeatedly and forcibly around its prey, while unsuccessfully attempting to eat it for nearly 7 h (Figure 4d), had a 33% reduction in circular prey diameter (from 6.9 to 4.6 cm; pre‐ingestion RPA = 137%).

We only observed coiling by nine individuals in 10 trials. The prey in these 10 trials was relatively large (RPA mean = 111%, range = 70%–149%), and several of these snakes that coiled were also large (SVL mean = 140 cm, range = 89–178 cm).

## 4 Discussion

### 4.1 Methods for Quantifying Relative and Maximal Prey Size

Size can have tremendous ontogenetic and interspecific variation, and size has profound consequences for the energy that animals require (Glazier 2005) and what animals are able to do, including during predator–prey encounters. Hence, diverse methods have been used to account for size, but which of the many metrics of size (e.g., length, diameter, cross‐sectional area, mass, etc.) is most appropriate depends on the question being asked. For gape‐limited predators such as snakes, one key question is simply whether the snake is big enough to eat prey, but another is what the energetic gain of eating prey is.

For understanding the feeding ecology of snakes, relative prey mass (RPM) has long been used (Fitch 1960; Greene 1983; Voris and Moffett 1981) and continues to be used widely (Glaudas et al. 2019; Kastner et al. 2024). RPM is very useful for gaining insights into the energy gained from prey (proportion to prey mass) relative to energetic requirement (proportional to predator mass) (Cundall and Greene 2000), which in turn can affect feeding frequency (Greene 1983; Greene and Wiseman 2023). However, RPM has limited utility for understanding the size of prey consumed relative to what is anatomically possible for the reasons that follow.

Many of the tissues such as fat and viscera that contribute the most to the mass of adult snakes (the denominator of RPM) have no causal relationship with gape, whereas several dimensions of the cranial bones are causally related to gape (Arnold 1983; Cundall and Irish 2022; Jayne 2023; King 2002). Consequently, significant correlations between snake mass, maximal gape, and maximal prey size can arise simply from the high correlations between mass and various metrics of skeletal size. Unfortunately, despite certain dimensions of the cranial bones being causally related to maximal gape, they can have surprisingly limited predictive value for maximal gape because of the vitally important and variable contribution to gape from the distension of the soft tissues (Figure 1) (Close et al. 2014; Jayne 2023; Petersen et al. 2024a). Thus, statistically significant correlations and relationships can occur and be quantified between many different aspects of snake size and potential maximal prey size, but some quantities such as RPM can be misleading as further illustrated by the following examples.

For snakes with similar SVL, the mass of snakes can vary considerably because of different amounts of fat and eggs or developing young. For example, for Burmese pythons caught in Florida, the heaviest snake was a gravid female (SVL = 488 cm) that weighed 95 kg, but the record SVL of 520 cm was a non‐gravid snake that weighed only 56 kg (Jayne et al. 2024). Consequently, for a given skeletal size of a snake and a given weight of prey, RPM could have a nearly two‐fold range in variation simply because of variation in snake mass associated with reproduction. However, for a given SVL, mass can also vary independently of the reproductive state of the snake, as occurs within a species for male snakes with varying amounts of fat or among different species that commonly have different amounts of muscle (Hoefer and Jayne 2013; Mathou et al. 2023).

**FIGURE 7:**
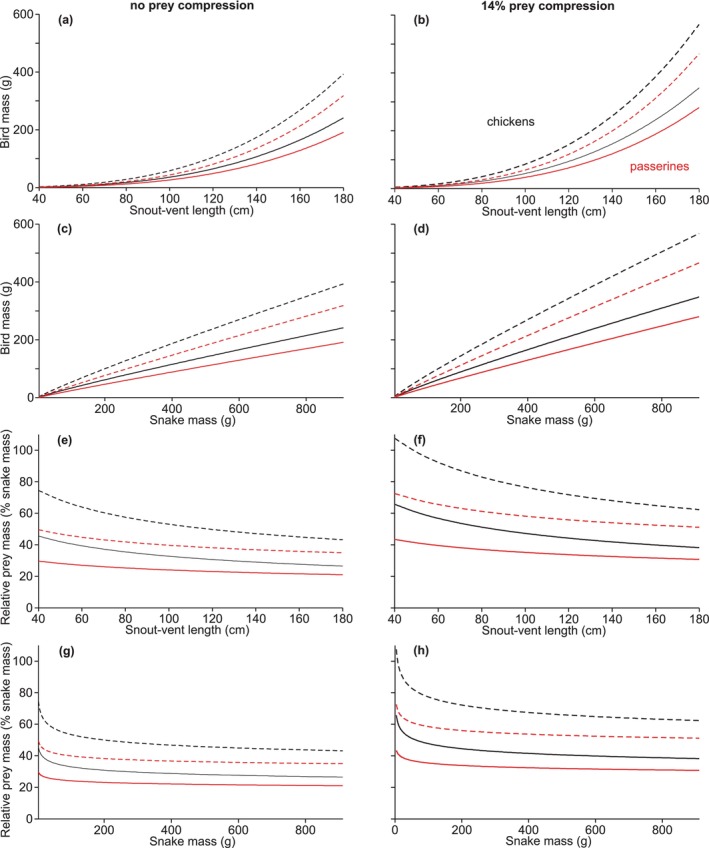
Limits on avian prey size predicted from the scaling relationships of maximal gape versus metrics of overall size of brown treesnakes and mass versus area for chickens (black) and passerine birds (red). Solid lines are values predicted from least‐squares scaling relationships of gape, and dashed lines are the upper 95% predication limits for those same regressions. The data in the left column assumed no compression of the prey during ingestion, whereas the values in the right column correct for a possible 14% reduction of prey diameter during ingestion. Despite larger snakes being able to eat prey with larger absolute size, the predicted relative prey masses (RPM) at maximal gape decrease substantially with increased overall snake size.

A value of RPM of 79% reported for a Såli (Micronesian starling) eaten by a brown treesnake with SVL = 116 cm and mass = 90 g (Kastner et al. 2024) could also be somewhat misleading regarding the maximal relative size of prey. If this was not simply a spurious value, then the snake involved was very emaciated as our scaling equations predict a mass of 188 g for SVL = 116 cm, which would more than halve the value of RPM for the same prey item. The second highest RPM reported for brown treesnakes eating a single Såli was 54% (Kastner et al. 2024), and unlike the previous value of RPM, this RPM is within the range although at the upper end of some of the predicted values for the species of birds used in our study (Figure 7e,f).

An implicit assumption of using RPM to gain insights in maximal prey size is that RPM for maximal size prey permitted by gape will be constant for all snake sizes within a species. However, none of the values of RPM predicted from maximal gape in our study (Figure 7) or in previous studies (Gripshover and Jayne 2021; Jayne 2023; Jayne et al. 2022) are constant for the entire range of snake sizes within a species. Instead, the combination of how gape scales with snake size and how prey mass scales with cross‐sectional area gives rise to complicated curvilinear relationships, for which the negative slopes of RPM versus snake mass and SVL change most rapidly for smaller snakes (Figure 7; Table 3). For example, the percentage decrease in predicted RPM from SVL of 50–100 cm (22.2%) is nearly twice that from 100 to 150 cm (13.9%). Such differences in the slope of RPM versus overall snake size are even greater when mass rather than SVL is used as overall snake size (Figure 7g vs. e).

An unexpected finding from our post‐ingestion dissections of prey was how much the cross‐sectional sizes of birds were reduced during feeding attempts, which allowed the snakes to eat prey with pre‐ingestion sizes that exceeded maximal gape. In retrospect, it would have been desirable to evaluate this post‐ingestion compression following all feeding trials. Even without coiling, the large, long‐term captives could reduce the pre‐ingestion diameters of birds and average of 14% with a range 6%–25%, and one failed attempt with vigorous coiling reduced bird diameter by 33%. Being able to consume prey with a 14% greater diameter may not seem like a sizable increase. However, since mass is proportional to the cross‐sectional area times length, a 14% increase in diameter can correspond to a 30% increase in cross‐sectional area and mass of prey with a given length. Given the considerable forces exerted when snakes kill their prey by constricting (Penning et al. 2015), perhaps some other snake species can also reduce the diameter of certain prey. Hence, some limitations to accurate predictions of maximal prey also exist even when both gape and prey size have been measured directly, but these are minor compared to those of RPM.

### 4.2 Prey Too Large to Consume

Feeding experiments can determine the size of prey too large to be eaten because of the limitations of gape only if the snakes are willing to attempt to eat prey as large as their gape permits. When different size prey are not eaten both within and among snake species, understanding whether this is caused by excessive prey size or other factors remains poorly understood because of the lack of direct measurements or estimates of gape from scaling equations.

At least for the recently caught brown treesnakes in our study, the refusal to attempt to eat prey (Figure 5c) was not very useful for gaining insights into the maximal size of prey that can be consumed because many prey that were refused (Figure 5c) had values of RPA well within the values that were commonly consumed by other snakes (Figure 5f). Hence, we suspect that many of these refusals for the snakes tested in Guam could have resulted from the snakes not being well adjusted to captivity, as none of the long‐term captives refused to eat any prey with RPA < 150%.

Another unexpected finding of our study was the willingness of brown treesnakes to attempt to eat birds that were vastly too large to swallow, including a maximum of RPA of 447% for a 1.25 kg chicken (Figure 4e). After the experiments in Guam revealed that the brown treesnakes often attempted to eat prey with values of RPA exceeding 150%, we expanded the range of prey sizes offered to the long‐term captives to try to establish what size was so large that no snake would attempt to eat it. However, we failed to find such an upper limit. Assuming the willingness to attempt to eat dead prey also reflects a willingness to attack live prey larger than can be consumed, this could markedly increase the potential effect of this predator on prey populations.

Additional evidence for the importance of accounting for attacks on prey too large to eat emerged from a recent field study of brown treesnakes in Guam (Kastner et al. 2024) that focused mainly on fledgling Såli (Micronesian starling, *Aplonis opaca* , Sturnidae, mass 60.5–86.3 g). Although we lacked direct measurements of the diameters of Såli, we did obtain these measurements for a closely related and similarly shaped species, the common starling ( *Sturnus vulgaris* , Sturnidae). The two common starlings had diameters, areas, and masses of 3.5 and 4.1 cm, 9.2 and 13.2 cm^2^, and 55 and 80 g, respectively (Table S1) nearly encompassing the range of masses for the Såli in the field study. The diameters for these two common starlings correspond to the predicted maximal gape of brown treesnakes with SVL of 115 and 141 cm, but the corresponding values of SVL are only 102 and 124 cm after allowing for a 14% reduction in diameter during the ingestion process. These values of SVL agree well with the finding that the smallest of the 55 brown treesnakes that ate a Såli fledgling had a SVL of 102 cm (Kastner et al. 2024), and the 73‐g fledgling eaten by this snake was near its limit for maximal edible prey size. Hence, the numerous partially eaten Såli fledglings found in Guam would likely have been killed by snakes with even smaller values of SVL than this.

Our observation of a long‐term captive attempting to eat a chicken with RPA of 447% provides additional insight into how small some of the brown treesnakes may have been that unsuccessfully tried to eat Såli. For example, if the 80‐g common starling had an RPA of 447% for a brown treesnake, that would correspond to a snake with SVL = 74 cm and mass = 37 g. An RPA of 447% for the smallest brown treesnake in our study (SVL = 40 cm, mass = 6.7 g) corresponds to a predicted diameter of 4.6 cm, which suggests that even the smallest snakes could pose a risk to the adults of numerous species of birds that formerly or currently occur on Guam and neighboring areas (Table 5; Table S2). Furthermore, only a tiny fraction of bird species on Guam and neighboring islands exceed the size of the largest chicken (diameter = 11.2 cm, mass = 1.25 kg) that a large, long‐term captive attempted to eat (Table S2). Three assumptions that are involved in these considerations of the snake sizes that could pose predation risks to birds remain to be tested. First, are at least some individuals of all size classes of brown treesnakes willing to attack and attempt to eat prey with similar values of RPA exceeding their gape? Second, are snakes either willing to attack or capable of subduing and killing live prey as large as dead prey that they attempted to eat in our experiments? Third, would laboratory feeding experiments with live prey accurately predict what snakes would do in the wild?

**TABLE 5:** Morphology of birds measured in Guam and its relationship with the size (SVL) of the snakes predicted to be able to eat birds if snakes used their maximal gape.

Species	Locale	*N*	Bird morphology						Predicted snake SVL (cm)			
			Mass (g)		Diam1 (cm)		Diam2 (cm)		Diam1		Diam2	
			Min	Max	Min	Max	Min	Max	Min	Max	Min	Max
Eurasian tree sparrow *Passer montanus*	G(I);C(I)	17	14.0	22	2.4	2.8	2.1	2.4	84	96	73	84
Black drongo *Dicrurus macrocercus*	G(I);C(I)	18	5.5	63	1.9	4.4	1.6	3.8	67	141	59	124
Siberian sand‐plover *Anarhynchus mongolus*	G;C	1	65		4.2		3.6		136		119	
Yellow bittern *Botaurus sinensis*	G;C	26	76	113	4.2	5.0	3.6	4.3	136	158	119	139
Pacific golden‐plover *Pluvialis fulva*	G;C	6	94	130	4.7	5.3	4.0	4.6	150	166	131	146
Black francolin *Francolinus francolinus*	G(I)	4	169	319	5.3	6.9	4.6	5.9	166	(209)	146	183
Rock pigeon *Columba livia*	G(I);C(I)	8	228	330	7.3	8.2	6.3	7.1	(219)	(242)	192	(213)

*Note:* Values of SVL in parentheses are highly improbable for this species of snake.

Abbreviations: C, Commonwealth of the Mariana Islands; Diam1, circular diameter of birds; Diam2, possible diameter of birds assuming a 14% reduction during ingestion; G, Guam; I, introduced; SVL, snout‐vent length of brown treesnakes.

For brown treesnakes and effectively all other species of snakes, much remains to be learned about the extent to which the size of live prey affects the probability of a predatory attack. For brown treesnakes in the field, the mass of live Såli fledglings did not significantly affect the probability of either consuming or attempting to consume birds (Kastner et al. 2024), whereas the sizes of the dead birds offered to the snakes in our study had highly significant effects on both of these probabilities. However, the range of masses in the field study (61–86 g) was small compared to the range of mass (28–1275 g) and RPA (17%–447%) used in our experiments.

One theoretical possibility is that snakes might almost indiscriminately attack potential prey, and then the sizes of prey that snakes attempt to eat and successfully eat are successively smaller subsets of the prey that were attacked as a result of not being large enough or formidable enough to escape being subdued or eaten. The most relevant data for testing generalities beyond our results for how commonly snakes within and among species attack prey too large to be swallowed are limited to five species of snakes that are crustacean specialists for which gape was measured directly. Field data (Gripshover and Jayne 2021; Jayne et al. 2018) found that two species that normally eat hard‐shelled crustaceans ( *Fordonia leucobalia* and *Liodytes alleni* ) did not eat any prey with RPA larger than 70%, whereas the two species that eat only freshly molted crustaceans ( *Gerarda prevostiana* , *Regina septemvittata* ) commonly ate prey with RPA near 100% or by tearing apart prey with an even greater value of RPA. For *F. leucobalia* in the laboratory, one‐third of the snakes attacked but were unsuccessful in subduing and eating hard‐shelled crabs with RPA from 129% to 462%, whereas all the snakes tested attacked and ate some portion of soft‐shelled crabs with similar size (Jayne et al. 2018). For another species only tested with laboratory feedings, none of the ten individuals of *Liodytes rigida* ate a crayfish with RPA exceeding 50%, but all these snakes attacked prey larger than the largest prey that was eaten (Gripshover and Jayne 2023). However, three of the *L. rigida* refused to attack the largest prey offered despite them being small enough to swallow (RPA 53%–95%). Collectively, these results show the complexity of how different snake species may have different predispositions to attack different prey of a given RPA, including sizes that are too large to be swallowed and how the defenses of the prey can winnow the range of prey sizes that are eaten compared to those of prey that are attacked.

### 4.3 Potential Costs of Large Prey

If snakes use an optimal feeding strategy, then one would expect that the potential benefits of attacking or eating prey should outweigh possible costs such as energy, time, and the risks of injury or death. As discussed elsewhere (Feder and Arnold 1982; Kastner et al. 2024), the energy expended by snakes during predation seems likely to be trivial compared to the energy gained from many prey items.

Whether increased ingestion times make snakes significantly more vulnerable to threats in the field is not obvious. Considering that less than 10 min was sufficient for our large captive snakes to consume a meal that could sustain them for 3–4 weeks, the ratio of these two times is certainly minuscule. However, we did observe some impressively long absolute times for ingestion by the brown treesnakes in our study. For example, the five longest successful feedings that we observed (RPA = 95%–129%) lasted from 95 to 102 min, and the five longest unsuccessful attempts (RPA 124%–365%) lasted from 60 to 417 min. Perhaps tradeoffs between risks and the probability of a benefit could help to explain why only 3 unsuccessful feedings lasted longer than the longest successful feeding and why the longest unsuccessful attempts were for prey with sizes near rather than greatly exceeding maximal gape.

Perhaps the most extreme risk during the feeding of snakes is that just eating or attempting to eat large prey can be fatal, as some evidence suggests that some snakes can swallow prey so large that it kills them. For example, a recent review of death following ingestion of 143 prey items by 73 species of snakes attributed nearly 41% of these deaths to the size of prey alone rather than fatal injuries resulting from sharp structures on the body of prey or prey defending themselves (Kornilev et al. 2022), and a significant number of these deaths included a complete rupture of the skin. None of the brown treesnakes in our study died after a feeding, but they were often euthanized within 24 h of being fed. However, the acellular layer of the skin in the neck ruptured in one of our feeding experiments with RPA = 95% (Figure 4b). Rupture of this layer prior to the normal time course during ecdysis can be fatal in some cases, and captive snakes commonly refuse to eat until ecdysis has been completed (Kauffeld 1969).

After ingestion, the added mass and change in snake shape can impede locomotion, but such effects depend on the size of the meal and the locomotor mode being used by the snake. For example, the rectilinear locomotion of boa constrictors is minimally affected even for meals as large as RPM = 37% (Petersen et al. 2024b), whereas the terrestrial lateral undulation of garter snakes is adversely affected after consuming meals with an average RPM of 22% (Garland Jr. and Arnold 1983). Unlike terrestrial locomotion on a continuous surface, arboreal locomotion on branches has risks of falling and toppling sideways. For brown treesnakes that were unfed or consumed meals of rodents averaging 12% and 21% RPM, feeding does adversely affect their arboreal locomotion but in a way that depends on meal size, the steepness of the cylindrical surface, and whether the cylinder is smooth or has secondary branches (Crotty and Jayne 2015). Besides the increased mass after feeding, the bulge of the prey item complicated the arboreal locomotion of the brown treesnakes. Because of differences in prey shape (Jayne et al. 2022), brown treesnakes would have a larger bulge after eating a bird compared to a rodent with the same mass. Nonetheless, and despite all these demonstrated or potential costs associated with large avian prey, the overall feeding strategy of brown treesnakes seems largely to conform to attempting to be a glutton when the opportunity presents itself.

## Author Contributions

**Shane R. Siers:** conceptualization (equal), data curation (equal), formal analysis (supporting), funding acquisition (lead), investigation (equal), methodology (equal), project administration (lead), writing – review and editing (equal). **Juan‐Carlos Mungaray:** data curation (supporting), investigation (supporting), writing – original draft (supporting). **Martin Kastner:** conceptualization (supporting), writing – review and editing (supporting). **Bruce C. Jayne:** conceptualization (equal), data curation (equal), formal analysis (lead), investigation (equal), methodology (equal), visualization (lead), writing – original draft (lead), writing – review and editing (lead).

## Conflicts of Interest

The authors declare no conflicts of interest.

## Supporting information


Appendix S1.

## Data Availability

The data that support the findings of this study are archived and publicly available at scholar@uc: https://scholar.uc.edu/show/sn00b040s.
